# Comparison of Preoperative Nutritional Indexes for Outcomes after Primary Esophageal Surgery for Esophageal Squamous Cell Carcinoma

**DOI:** 10.3390/nu13114086

**Published:** 2021-11-15

**Authors:** Jung-Pil Yoon, Jae-Sik Nam, Mohd Fitry Bin Zainal Abidin, Seon-Ok Kim, Eun-Ho Lee, In-Cheol Choi, Ji-Hyun Chin

**Affiliations:** 1 Department of Anesthesiology and Pain Medicine, Asan Medical Center, University of Ulsan College of Medicine, Seoul 05505, Korea; wizdumb@naver.com (J.-P.Y.); jaesik_nam@naver.com (J.-S.N.); icchoi@amc.seoul.kr (I.-C.C.); 2 Department of Anesthesia and Intensive Care, University Malaya Medical Centre, Petaling Jaya 59100, Malaysia; mohdfitry@yahoo.co.uk; 3 Asan Medical Center, Department of Clinical Epidemiology and Biostatistics, University of Ulsan College of Medicine, Seoul 05505, Korea; seonok@amc.seoul.kr; 4 Department of Anesthesiology and Pain Medicine, University of Ulsan College of Medicine, Seoul 05505, Korea; leho@naver.com

**Keywords:** esophageal cancer, controlling nutritional status score, prognostic nutritional index, geriatric nutritional risk index, survival

## Abstract

Background: This study aimed to compare the controlling nutritional status (CONUT) score, prognostic nutritional index (PNI), and geriatric nutritional risk index (GNRI) for predicting postoperative outcomes in patients with esophageal squamous cell carcinoma undergoing esophagectomy. Methods: We retrospectively reviewed the data of 1265 consecutive patients who underwent elective esophageal surgery. The patients were classified into no risk, low-risk, moderate-risk, and high-risk groups based on nutritional scores. Results: The moderate-risk (hazard ratio [HR]: 1.55, 95% confidence interval [CI]: 1.24–1.92, *p* < 0.001 in CONUT; HR: 1.61, 95% CI: 1.22–2.12, *p* = 0.001 in GNRI; HR: 1.65, 95% CI: 1.20–2.26, *p* = 0.002 in PNI) and high-risk groups (HR: 1.91, 95% CI: 1.47–2.48, *p* < 0.001 in CONUT; HR: 2.54, 95% CI: 1.64–3.93, *p* < 0.001 in GNRI; HR: 2.32, 95% CI: 1.77–3.06, *p* < 0.001 in PNI) exhibited significantly worse 5-year overall survival (OS) compared with the no-risk group. As the nutritional status worsened, the trend in the OS rates decreased (*p* for trend in all indexes < 0.05). Conclusions: Malnutrition, evaluated by any of three nutritional indexes, was an independent prognostic factor for postoperative survival.

## 1. Introduction

Esophageal cancer is the seventh most common type of malignancy and the sixth leading cause of cancer-related death worldwide, with esophageal squamous cell carcinoma (ESCC) being the main histological type in Asian countries [1]. Patients with esophageal cancer are often malnourished at diagnosis, and malnutrition is associated with poor prognosis [2]. Although the prevalence of hospital malnutrition is as high as approximately 20–50%, its importance is frequently underestimated in clinical practice due to the lack of acknowledgement, as well as the lack of a standard nutritional risk screening tool [3,4].

Currently, nutritional assessment indexes, including the controlling nutritional status (CONUT) score [5], geriatric nutritional risk index (GNRI) [6], and prognostic nutritional index (PNI) [7,8] have been developed, and are used to assess the influence of nutritional status on the prognosis of esophageal cancer. These indexes use different combinations of serum albumin level, peripheral total lymphocyte counts, total cholesterol levels, and anthropometric factors, such as body mass index, which are associated with nutrition and cancer progression. A high CONUT score, low PNI, and low GNRI were prognostic factors for postoperative survival in patients with esophageal cancer [9,10,11,12,13,14,15], although discrepancies were observed in previous studies on postoperative morbidities [12,16,17,18]. To the best of our knowledge, no study has compared the ability of these indexes to concurrently predict postoperative long-term and short-term outcomes after esophageal cancer surgery. The identification of simple, objective, and easily accessible nutritional screening tools would not only help to predict postoperative outcomes, but also assist in selecting appropriate perioperative nutritional management for esophageal cancer. This study aimed to evaluate preoperative nutrition-related risks using the CONUT score, PNI, and GNRI, and compare the three indexes to identify prognostic values for postoperative outcomes after primary esophageal cancer surgery.

## 2. Materials and Methods

### 2.1. Study Design and Participants

This retrospective cohort study included patients aged ≥ 20 years who underwent elective esophageal surgery at a tertiary hospital between January 2005 and December 2018. All clinical data were obtained from the Asan Medical Center Esophageal Surgery and Anesthesia Database and by a retrospective review of the computerized patient record system (Asan Medical Center Information System Electronic Medical Record) [19]. We excluded patients with esophagus tumors other than ESCC; those who underwent repeat surgery, non-esophagectomy, or other surgeries simultaneously; and those lacking preoperative laboratory test results (i.e., serum albumin, lymphocyte, and total cholesterol). This study was performed in accordance with the Strengthening the Reporting of Observational Studies in Epidemiology guidelines [20] and was approved by the Institutional Review Board of Asan Medical Centre (protocol number: 2020-1804), which waived the requirement for informed consent.

### 2.2. Calculation of Preoperative Nutritional Status

Preoperative nutritional status was assessed using the CONUT score, GNRI, and PNI. The CONUT score was calculated by adding the scores of the following parameters: serum albumin level [≥3.5 g/dL (0 points), 3.0–3.4 g/dL (2 points), 2.5–2.9 g/dL (4 points), or <2.5 g/dL (6 points)], total lymphocyte count [≥1600 cells/μL (0 points), 1200–1599 cells/μL (1 points), 800–1199 cells/μL (2 points), or <800 cells/μL (3 points)], and total cholesterol level [≥180 mg/dL (0 point), 140–179 mg/dL (1 point), 100–139 mg/dL (2 points), or <100 mg/dL (3 points)] [5]. The GNRI was calculated using the following equation: 14.89 × serum albumin level (g/dL) + 41.7 × (present body weight/ideal body weight). The ideal body weight was calculated using the following Lorenz equation: height − 100 − [(height − 150)/4] for men, and height − 100 − [(height − 150)/2.5] for women [6]. The units were expressed as kilograms (kg; weight) and centimeters (cm; height). The PNI was calculated as 10 × serum albumin level (g/dL) + 0.005 × total lymphocyte count (cells/μL) [21]. Preoperative blood samples measured closest to the time of surgery (but within 1 month of surgery) were used to calculate the nutritional parameters.

The patients were classified into no-risk (CONUT: 0–1, GNRI: >98, PNI: >50), low-risk (CONUT: 2, GNRI: 92 to ≤98, PNI: 44.16 to ≤50), moderate-risk (CONUT: 3–4, GNRI: 82 to <92, PNI: 42 to <44.16), and high-risk groups (CONUT: ≥5, GNRI: <82, PNI: <42), as determined by our preliminary analysis including the receiver operating characteristic (ROC) curve analysis and previous studies [5,6,13,21,22,23]. Malnutrition (CONUT: ≥3, GNRI: <92, PNI: <44.16) was defined as a moderate to high nutritional risk categorized using each method.

### 2.3. End Points

The primary end point of the study was overall survival (OS) after surgery. OS was calculated as the period from the date of surgery to the date of death from any cause or last follow-up. The secondary outcomes were recurrence-free survival (RFS) and the presentation of composite major complications within 30 days after surgery. RFS was calculated from the date of surgery to either the recorded day of initial recurrence or the date of death or last follow-up. Postoperative cancer recurrence was defined as a radiological/histological diagnosis of recurrence. Data regarding death and cancer recurrence were obtained from outpatient clinics, through a detailed review of medical records and telephone interviews, or from the National Population Registry of the Korean National Statistical Office. The last evaluation of survival status was performed in August 2020. A postoperative 30-day composite major complication was defined as a composite outcome of any one or more of the following complications: (1) all-cause death, (2) major adverse cardio-cerebrovascular events (myocardial infarction, malignant ventricular arrhythmia, cardiac dysfunction, and ischemic or hemorrhagic stroke), (3) respiratory complications (respiratory failure requiring mechanical ventilation for more than 48 h or reintubation, pneumonia, or acute respiratory distress syndrome), (4) wound or infectious complications (wound infection, anastomosis leak, or sepsis), (5) renal complications (≥Kidney Disease Improving Global Outcomes stage 2 or requirement for renal replacement therapy), and (6) multi-organ failure. A patient experiencing more than one single event was counted only once in the composite outcome. The major postoperative complications were defined according to the European Perioperative Clinical Outcome definitions, or as previously reported [24,25]. The esophageal cancer pathologic stage was determined using the TNM classification from the 7th edition of the American Joint Committee on Cancer.

### 2.4. Statistical Analysis

A priori power analysis was not conducted, and the study sample size was determined by all patients included in the study. Continuous variables were expressed as the mean ± standard deviation or medians with interquartile range, whereas categorical variables were expressed as numbers and percentages. Between-group differences were evaluated using the Student’s *t* test or Mann–Whitney test for continuous variables, and the chi-squared test or Fisher’s exact test for categorical variables, as appropriate.

The correlations and agreements between nutritional scores calculated using the three equations were assessed by Spearman’s correlation analyses and weighted kappa statistic. ROC analyses were performed, and the results are presented as adjusted areas under the ROC curves (AUCs) with 95% confidence intervals (CIs) to evaluate the sensitivity and specificity for predicting OS.

Univariate and multivariable Cox proportional hazard regression models were used to identify potential prognostic factors for OS and RFS. The proportional hazards assumption was confirmed by the examination of log (−log [survival]) curves and by testing of the partial (Schoenfeld) residuals; no relevant violations were found.

The variables in Table 1 were tested; variables with a *p* value of <0.20 in the univariate analyses were included in the multivariable analyses. The missing values were replaced by imputed values using the Markov chain Monte Carlo method. The final model was determined using the backward elimination process. The *p* for trend test using Cox regression analysis was performed to investigate OS trends across the four nutritional status levels. To estimate the effects of nutritional status according to the pathologic cancer stage, the interaction term between nutritional status and pathologic stage was included in the multivariable model. Survival probability was estimated using the Kaplan–Meier method; differences in survival were evaluated using a log-rank sum test, and Bonferroni correction was used as the post hoc test. Univariate and multivariable logistic regression analyses were performed to assess the potential factors for predicting postoperative composite complications.

All reported *p* values were two-sided, and *p* values of <0.05 were considered significant. All data manipulations and statistical analyses were performed using SAS^®^ version 9.4 (SAS Institute Inc., Cary, NC, USA) software and IBM SPSS Statistics 25.0 (IBM Corp., Armonk, NY, USA).

## 3. Results

**Figure 1:**
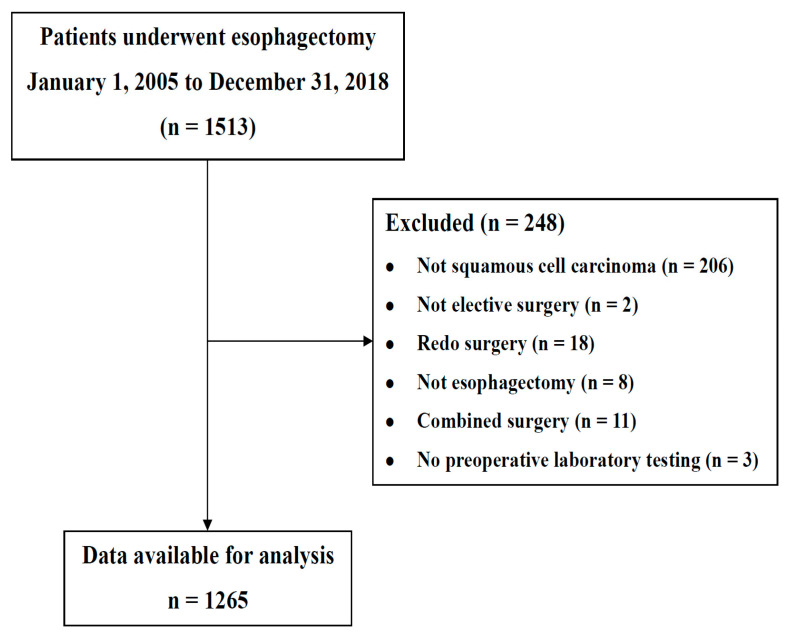
Study inclusion/exclusion flow diagram.

Among 1513 patients, 1265 were eligible for inclusion in the present study (Figure 1).

Baseline and perioperative characteristics of these patients are shown in Table 1.

The average age was 63.0 (57.5–69.0) years, and 6.7% of the patients were women. The follow-up period was 44.0 (24.0–89.0) months.

**Figure 2:**
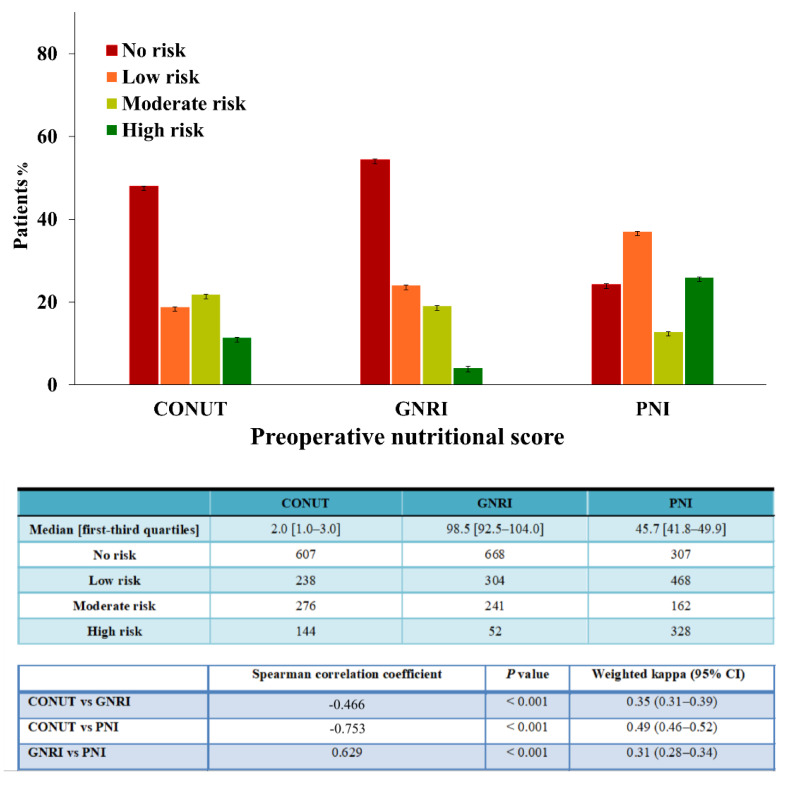
Distribution of patients by nutritional status and correlation and agreements between each pair of nutritional scores calculated by the three scoring methods.

Figure 2 shows the distribution of patients into different categories based on their nutritional status, and the correlation and weighted kappa statistics for the three nutritional indexes.

The correlation analyses showed a significant correlation between the three nutritional indexes. When the patients were classified into four groups, a fair agreement was observed between the three nutritional indexes.

The prognostic accuracies of the CONUT score, GNRI, and PNI were explored using the AUC of the ROC curve for predicting the OS. The AUCs of the CONUT score, GNRI, and PNI for OS were 0.624 (95% CI: 0.593–0.655), 0.633 (95% CI: 0.603–0.664), and 0.628 (95% CI: 0.597–0.659), respectively.

The postoperative outcome data are shown in Table 2.

Regardless of the method used to assess nutritional status, intensive care unit stay and hospital stay after surgery were significantly longer in patients with malnutrition than in those without malnutrition. The incidences of postoperative 30-day composite complications, 90-day death, and 1-year death were also higher in patients with malnutrition. In the multivariable analyses, malnutrition by GNRI and PNI, but not by CONUT score, was associated with an increased risk of postoperative 30-day composite complications (Table S1).

**Figure 3:**
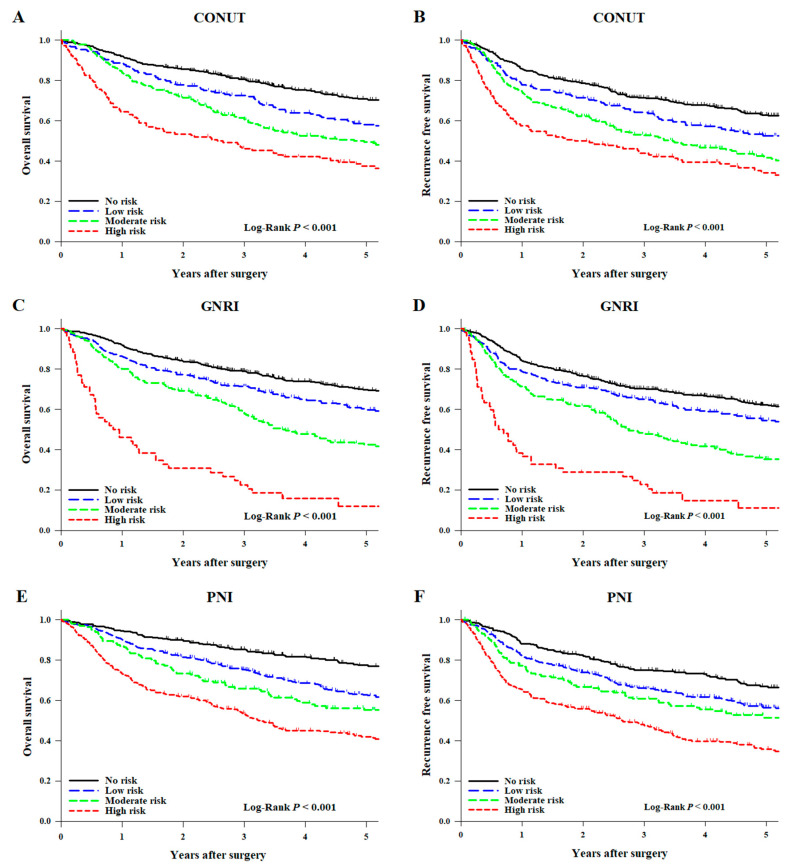
Kaplan–Meier analysis of (**A**,**C**,**E**) overall survival and (**B**,**D**,**F**) recurrence-free survival in groups of patients assorted by preoperative nutritional scores. The patients were classified into no risk (CONUT: 0–1, GNRI: >98, PNI: >50), low-risk (CONUT: 2, GNRI: 92 to ≤98, PNI: 44.16 to ≤50), moderate-risk (CONUT: 3–4, GNRI: 82 to <92, PNI: 42 to <44.16), and high-risk groups (CONUT: ≥5, GNRI: <82, PNI: <42). CONUT, controlling nutritional status; GNRI, geriatric nutritional risk index; PNI, prognostic nutritional index.

The Kaplan–Meier curve showed that the 5-year OS rates decreased from the lower nutritional risk group to the higher nutritional risk group in all three assessment methods [CONUT = no risk: 70.6% (95% CI: 66.6–74.3), low risk: 58.1% (95% CI: 51.0–64.5), moderate risk: 49.3% (95% CI: 42.8–55.5), and high risk, 37.5% (95% CI: 29.3–45.7) (*p* < 0.001, Figure 3A); GNRI = no risk: 69.6% (95% CI: 65.7–73.1), low risk: 59.8% (95% CI: 53.6–65.4), moderate risk: 42.4% (95% CI: 35.6–49.1), and high risk: 11.9% (95% CI: 4.1–24.2) (*p* < 0.001, Figure 3C); PNI = no risk: 77.0% (95% CI: 71.4–81.5), low risk: 62.4% (95% CI: 57.5–67.0), moderate risk: 55.3% (95% CI: 46.8–63.1), and high risk: 41.9% (95% CI: 36.2–47.6) (*p* < 0.001, Figure 3E)]. The 5-year RFS rates were also lower in patients with poor preoperative nutritional status (Figure 3B for CONUT; Figure 3D for GNRI; Figure 3F for PNI).

In the multivariable analyses (Table 3), compared with the no-risk group, the moderate-risk group (HR: 1.55, 95% CI: 1.24–1.92, *p* < 0.001 in CONUT; HR: 1.61, 95% CI: 1.22–2.12, *p* = 0.001 in GNRI; HR: 1.65, 95% CI: 1.20–2.26, *p* = 0.002 in PNI) and the high-risk group (HR: 1.91, 95% CI: 1.47–2.48, *p* < 0.001 in CONUT; HR: 2.54, 95% CI: 1.64–3.93, *p* < 0.001 in GNRI; HR: 2.32, 95% CI: 1.77–3.06, *p* < 0.001 in PNI) were associated with worse OS.

Other variables associated with the OS are shown in Tables S2–S4. Compared with the no-risk group, the moderate-risk group (HR: 1.40, 95% CI: 1.45–1.72, *p* = 0.001 in CONUT; HR: 1.38, 95% CI: 1.07–1.79, *p* = 0.015 in GNRI; HR: 1.40, 95% CI: 1.05–1.88, *p* = 0.023 in PNI) and the high-risk group (HR: 1.65, 95% CI: 1.29–2.11, *p* < 0.001 in CONUT; HR: 2.03, 95% CI: 1.33–3.09, *p* = 0.001 in GNRI; HR: 1.90, 95% CI: 1.48–2.44, *p* < 0.001 in PNI) were associated with worse RFS. In all nutritional status assessment tools, as the preoperative nutritional status worsened, the OS (*p* for trend < 0.001 in CONUT, GNRI, and PNI) and RFS rates decreased (*p* for trend < 0.001 in CONUT and PNI; *p* for trend = 0.001 in GNRI).

**Figure 4:**
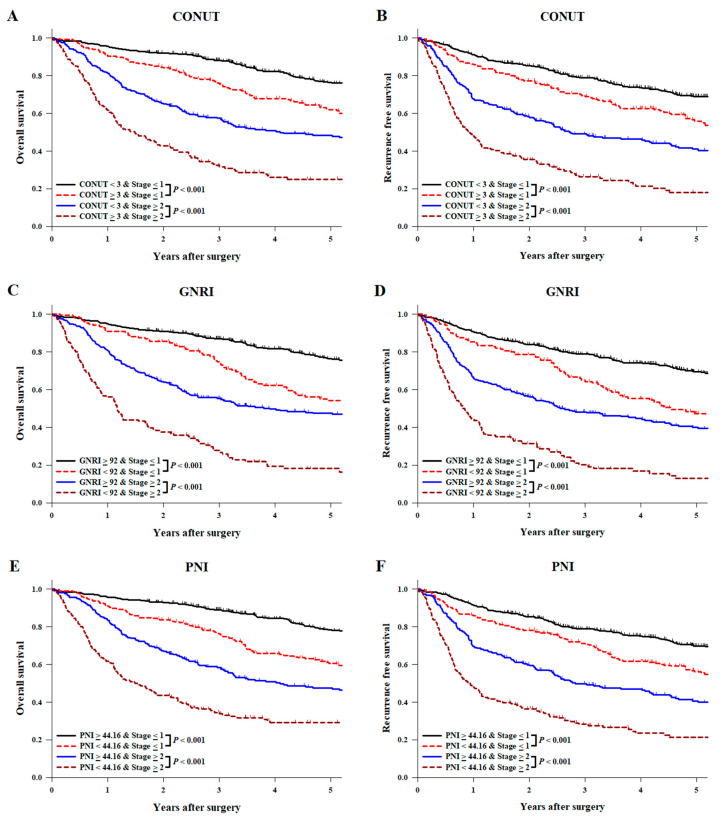
Kaplan–Meier analysis of (**A**,**C**,**E**) overall and (**B**,**D**,**F**) recurrence-free survival curves stratified with the three nutritional assessment methods and pathologic stage. CONUT, controlling nutritional status; GNRI, geriatric nutritional risk index; PNI, prognostic nutritional index.

Results of the comparison of OS and RFS stratified according to pathologic stage are shown in Figure 4.

In both low (stage ≤ 1) and high (stage ≥ 2) pathologic stages, both survival rates were lower in the malnutrition group compared with the normal group [5-year OS rate = CONUT: 61.9% (95% CI: 54.8–68.3) vs. 76.1% (95% CI: 72.0–79.6) in the low stage, *p* < 0.001, whereas 23.7% (95% CI: 17.2–30.9) vs. 47.7% (95% CI: 41.3–53.8) in the high stage, *p* < 0.001, Figure 4A; GNRI: 54.1% (95% CI: 45.1–62.3) vs. 76.2% (95% CI: 72.5–79.6) in the low stage, *p* < 0.001, whereas 16.4% (95% CI: 9.9–24.3) vs. 46.9% (95% CI: 41.1–52.6) in the high stage, *p* < 0.001, Figure 4C; PNI: 60.5% (95% CI: 54.1–66.4) vs. 78.0% (95% CI: 73.9–81.6) in the low stage, *p* < 0.001, whereas 28.0% (95% CI, 21.5–34.8) vs. 46.9% (95% CI, 40.2–53.4) in the high stage, *p* < 0.001, Figure 4E; 5-year RFS rates = CONUT: 55.8% (95% CI: 48.5–62.6) vs. 68.9% (95% CI: 64.6–72.9) in the low stage, *p* < 0.001, whereas 18.1% (95% CI: 12.1–25.2) vs. 40.4% (95% CI: 33.9–46.8) in the high stage, *p* < 0.001, Figure 4B; GNRI: 47.2% (95% CI: 38.2–55.8) vs. 69.4% (95% CI: 65.3–73.1) in the low stage, *p* < 0.001, whereas 13.0% (95% CI: 7.4–20.4) vs. 39.3% (95% CI: 33.4–45.3) in the high stage, *p* < 0.001, Figure 4D; PNI: 56.1% (95% CI: 49.4–62.3) vs. 69.9% (95% CI: 65.3–73.9) in the low stage, *p* < 0.001, whereas 21.5% (95% CI: 15.3–28.3) vs. 39.9% (95% CI: 33.1–46.6) in the high stage, *p* < 0.001, Figure 4F].

The effect of preoperative nutritional status evaluated by CONUT and PNI on OS was not dependent on pathologic stage (interaction *p* = 0.546 in CONUT; interaction *p* = 0.193 in PNI). However, the effect of preoperative nutritional status evaluated by GNRI on OS was more significant at a high pathologic stage (interaction *p* = 0.047). The effect of preoperative nutritional status on RFS was not dependent on pathologic stage (interaction *p* = 0.328 in CONUT; interaction *p* = 0.169 in GNRI; interaction *p* = 0.191 in PNI).

## 4. Discussion

In this study, the CONUT score, PNI, and GNRI were compared in terms of their prognostic ability for short-term and long-term postoperative outcomes in 1265 patients with ESCC who underwent esophagectomy. The main findings were as follows: (1) a high CONUT score, low PNI, and low GNRI were significantly associated with a worse 5-year OS and RFS after esophageal surgery for ESCC; (2) the survival rate tended to worsen as the nutritional status progressed from moderate risk to high risk; and (3) although the PNI and the GNRI showed prognostic value for postoperative 30-day composite complications, the CONUT score did not.

In esophageal cancer patients, the prognostic role of nutritional indexes, including the CONUT score, PNI, and GNRI on the postoperative outcomes, has been reported previously. One study reported that a high CONUT score (≥5) predicts poor prognosis in patients who underwent esophagectomy for esophageal cancer [26]. Other studies reported that the CONUT score has more significant predictive power for postoperative survival in esophageal cancer patients compared with inflammatory biomarkers, including the neutrophil-to-lymphocyte ratio or platelet-to-lymphocyte ratio [10,15]. A series of meta-analyses were conducted in PNI studies, which confirmed the prognostic value of low PNI on worse OS and RFS in patients who underwent esophagectomy [13,18,27]. Lastly, low GNRI (GNRI < 92) was a useful independent prognostic factor for 5-year OS in ESCC patients who underwent esophagectomy [12,17]. These previous studies were either conducted with relatively small study samples or evaluated only the morbidity or mortality rates. Therefore, our study has the following clinical implication: ours was a large cohort study that aimed to evaluate both short-term and long-term outcomes. In line with previous studies, this study revealed the predictive value of all three indexes on long-term mortality, suggesting that any of the three indexes can potentially be used as a screening tool for preoperative nutrition-related risk evaluation in patients who undergo esophageal surgery. Compared with the no-risk group, the group with worse nutritional status exhibited a poorer prognosis, implicating that severity, as well as malnutrition itself, are important factors for the prognosis of esophageal surgery. However, for the evaluation of short-term morbidities, the CONUT score did not show predictive power in our study. Although previous survival studies have shown relatively consistent results regarding the prognostic value of malnutrition evaluated by any of the three indexes [10,11,12,13,14,15,17,18], the results of previous studies related to postoperative complications were not comprehensive [12,14,16,28,29,30]. These inconsistencies may be because the method used to determine the optimal cut-off value was not standardized. In our study, the cut-off values for CONUT and PNI were set using an ROC curve method; the cut-off value for GNRI was determined based on the results of Bouillanne et al.’s study [6]. Further studies are needed to establish the optimal cut-off values to increase the reliability and accuracy of the study results.

Malnutrition is a poor prognostic factor of postoperative mortality in various cancer types, because nutrition affects cancer progression and the therapeutic responses of various malignancies [2,31,32]. To assess the influence of malnutrition-related risk on postoperative outcomes, indexes include nutritional, inflammatory, and immunological parameters. Low serum albumin levels have traditionally been considered as a biomarker of protein reserves and nutritional status [33]. Serum albumin is also closely related to systemic inflammation in patients with cancer. Inflammatory cytokines surge as tumor cells progress, contributing to the suppression of albumin synthesis, albumin degradation, and the capillary escape of albumin [34]. Therefore, serum albumin reflects nutritional status and systemic inflammation. Lymphocytes mediate anticancer responses and indicate cell-mediated immunological status [35]. Total cholesterol levels indicate the caloric reserves [36]. In addition, weight loss is a characteristic of esophageal cancer due to dysphagia and poor intake caused by tumor obstruction, resulting in poor survival [2,37]. Although a significant correlation was found between the three indexes, different parameters in each index might have influenced the prevalence of malnutrition, with a remarkable prevalence of malnutrition observed based on the PNI scores. The PNI only includes two biochemical markers; therefore, it could have classified more patients as malnourished. This finding is clinically meaningful, corroborating a previous study, which also demonstrated that PNI included more patients classified as malnourished compared with the CONUT and GNRI in esophageal cancer [38].

Several previous studies have identified the optimal nutritional index. Wang et al. reported that the GNRI shows better consistency with malnutrition diagnostic criteria than the CONUT, PNI, and NRI [38]. In contrast, Yoshida et al. mentioned that the CONUT score is not a reliable marker of malnutrition because the parameters included in this index are affected by other factors irrelevant to nutrition, such as liver function or dehydration [26]. In our study, the GNRI was slightly more associated with postoperative OS than the other two indexes. A possible explanation for the better prediction over others is that the GNRI is a multi-dimensional index, which accounts for weight loss, the main mechanism of malnutrition in patients with esophageal cancer. In addition, it seems that the GNRI does not only indicate the nutritional status, but also reflects cancer progression. A previous study reported that a larger primary tumor size and higher incidence of preoperative dysphagia were observed in the low-GNRI group among those with stage Ⅲ ESCC [12]. In our subgroup analysis with patients stratified according to pathologic stage, the effect of nutritional status evaluated by the GNRI on OS was more significant at a high pathologic stage, although CONUT and PNI did not exhibit significantly different interactions by pathologic stage. Although the underlying mechanism is unknown, locally advanced cancer extends beyond the mucosal layer, and tumor obstruction in the esophagus results in dysphagia and further exacerbates weight loss, which may have been reflected in the GNRI [33].

Regular assessments of nutrition-related risk and optimizations of preoperative nutritional status are the goals of perioperative nutritional management. The European Society for Clinical Nutrition and Metabolism (ESPEN) guidelines highly recommend nutritional support 10–14 days prior to major surgery in patients presenting with severe nutritional risk [39]. Recently, several studies have explored potential nutritional targets for patients at high risk of malnutrition, using preoperative carbohydrate treatment, vitamin D supplementation, hemoglobin optimization, and immune nutrition [40,41,42]. However, current evidence is limited to recommend their routine use [40]. One prospective analysis study reported that the administration of exogenous ghrelin during chemotherapy in esophageal cancer improved the nutritional status and significantly reduced adverse events [43]. Further studies are needed to validate the actual benefits of preoperative nutritional support in ESCC patients with malnutrition using the CONUT, PNI, and GNRI.

Our study has several limitations. First, we cannot exclude other confounding factors beyond those examined, and these factors might be associated with the nutritional status or survival. Second, there are no standard cut-off values for each index, resulting in inaccuracies in screening and treatment planning. Further studies are needed to establish universally accepted cut-off values to apply these nutritional indexes as screening tools and use them in selecting an appropriate perioperative nutritional treatment. Third, this study included only Korean patients with ESCC. It remains uncertain whether our results can be directly applied to patient groups with other histologic types of esophageal cancer, such as Western populations with adenocarcinoma as the most common histologic subtype. Although this study did not reveal the effect of nutritional support in patients with malnutrition on postoperative prognosis, our results have a strength in that preoperative identification of patients in malnutrition evaluated by any of the three nutritional indexes may provide information to predict postoperative mortality in ESCC. All three indexes are practical and affordable to be used as malnutrition screening tools.

## 5. Conclusions

The CONUT score, PNI, and GNRI are objective evaluation methods that enable quantitative assessment of the nutrition-related risk of mortality in esophageal cancer surgery. Malnutrition evaluated using the three nutritional indexes can be used as a therapeutic target to reduce the potential mortality risk in perioperative patients with ESCC.

## Figures and Tables

**Table 1:** Baseline and perioperative characteristics of the patient population.

Variables		Univariate Analysis for Overall Survival	
N	1265 (100)	Hazard Ratio (95% CI)	*p* Value
Baseline characteristics			
Age (years)	63.0 [57.5–69.0]	1.02 (1.01–1.04)	<0.001
Female	85 (6.7)	0.94 (0.67–1.31)	0.712
Body mass index (kg/m^2^)	23.1 ± 3.0	0.88 (0.86–0.91)	<0.001
ASA class			0.112 †
I	88 (7.0)	reference	
II	1122 (88.7)	1.12 (0.81–1.53)	0.501
III	55 (4.3)	1.63 (1.01–2.63)	0.048
Hct (%)	38.5 [34.8–41.4]	0.92 (0.90–0.93)	<0.001
Creatinine (mg/dL)	0.82 [0.71–0.94]	0.84 (0.58–1.24)	0.383
Bilirubin, total (mg/dL)	0.5 [0.4–0.7]	0.75 (0.55–1.02)	0.067
Albumin (g/dL)	3.7 [3.5–4.0]	0.36 (0.29–0.45)	<0.001
Uric acid (mg/dL)	5.2 [4.4–6.2]	0.87 (0.82–0.92)	<0.001
Lymphocyte count (cells/μL)	1698 [1172–2215]	1.00 (0.99–1.00)	<0.001
Total cholesterol (mg/dL)	174 [149–197]	0.99 (0.98–0.99)	<0.001
LVEF (%)	62 [59–65]	0.99 (0.97–1.01)	0.290
FVC (% predicted)	92.0 [84.0–100.0]	0.99 (0.98–0.99)	0.006
FEV_1_ (% predicted)	92.0 [82.0–100.3]	0.99 (0.99–1.00)	0.018
FEV_1_/FVC	74.0 [68.0–78.0]	0.99 (0.99–1.01)	0.310
Diabetes mellitus	193 (15.3)	1.37 (1.12–1.69)	0.003
Hypertension	459 (36.3)	0.99 (0.84–1.17)	0.906
Cerebrovascular disease	40 (3.2)	1.04 (0.67–1.63)	0.857
COPD	27 (2.1)	1.82 (1.14–2.92)	0.012
Chronic kidney disease	49 (3.9)	1.54 (1.08–2.19)	0.016
Liver disease	97 (7.7)	1.01 (0.75–1.37)	0.932
Smoking status			0.155 †
Non-smoking	388 (30.7)	reference	
Ex-smoking	609 (48.1)	1.21 (0.99–1.47)	0.056
Current smoking	268 (21.2)	1.11 (0.88–1.41)	0.371
Alcohol	940 (74.3)	1.09 (0.89–1.34)	0.405
Chemo-radiation therapy	474 (37.5)	1.88 (1.60–2.20)	<0.001
ACEI or ARB	240 (19.0)	0.73 (0.59–0.92)	0.006
β-blocker	245 (19.4)	0.92 (0.72–1.17)	0.491
Calcium channel blocker	249 (19.7)	0.83 (0.67–1.03)	0.084
Diuretics	108 (8.5)	1.05 (0.80–1.39)	0.719
Insulin	190 (15.0)	1.37 (1.11–1.69)	0.004
Oral hypoglycemic agent	135 (10.7)	1.41 (1.11–1.79)	0.005
Statins	161 (12.7)	1.13 (0.88–1.44)	0.337
Perioperative data			
Anesthesia time (hours)	6.8 [5.6–8.0]	1.11 (1.06–1.16)	<0.001
Crystalloid (L)	1.7 [1.2–2.2]	1.19 (1.10–1.28)	< 0.001
Colloid (L)	0.6 [0.1–1.0]	1.05 (0.89–1.23)	0.598
Use of pRBC *	206 (16.3)	2.17 (1.80–2.61)	< 0.001
Ivor Lewis	581 (45.9)	0.81 (0.69–0.96)	0.012
Minimally invasive surgery	385 (30.4)	0.72 (0.59–0.88)	0.001
Weight gain (%)	0.9 [−0.2–2.3]	1.04 (1.01–1.07)	0.035
Immediate postoperative Hct (%)	36.0 [32.0–39.5]	0.92 (0.90–0.93)	<0.001
Maximal SOFAc score	0 [0–2]	1.32 (1.24–1.40)	<0.001
Pathologic stage of cancer			<0.001 †
0	238 (18.8)	reference	
I	562 (44.4)	0.76 (0.60–0.96)	0.021
II	248 (19.6)	1.81 (1.42–2.32)	<0.001
III	204 (16.1)	3.79 (2.95–4.89)	<0.001
IV	13 (1.0)	3.70 (1.99–6.89)	<0.001

Data are expressed as number of patients (%), mean ± standard deviation, or median [first-third quartiles]. * used intraoperatively and postoperatively. †: The *p* values are the overall *p* value of the corresponding variables. ASA, American Society of Anesthesiology; LVEF, left ventricle ejection fraction; FVC, forced vital capacity; FEV_1_, forced expiratory volume in 1 s; COPD, chronic obstructive pulmonary disease; ACEI, angiotensin-converting enzyme inhibitor; ARB, angiotensin receptor blocker; pRBC, packed red blood cell; Hct, Hematocrit; SOFAc, cardiovascular sequential organ failure assessment in the first 24 h.

**Table 2:** Postoperative complications according to preoperative nutritional status.

	CONUT		*p* Value	GNRI		*p* Value	PNI		*p* Value
	Normal	Malnutrition		Normal	Malnutrition		Normal	Malnutrition	
	≤2	≥3		≥92	<92		≥44.16	<44.16	
N	845 (66.8)	420 (33.2)		972 (76.8)	293 (23.2)		775 (61.3)	490 (38.7)	
ICU stay (d)	1.0 [0.8–1.8]	1.0 [0.9–1.9]	0.005	1.0 [0.8–1.8]	1.0 [0.9–1.9]	0.003	1.0 [0.8–1.8]	1.0 [0.9–1.9]	0.007
Hospital stay (d)	13 [11–16]	15 [12–18]	<0.001	13 [11–16]	15 [12–21]	<0.001	13 [11–16]	14 [12–18]	<0.001
MACCE	22 (2.6)	21 (5.0)	0.040	27 (2.8)	16 (5.5)	0.042	20 (2.6)	23 (4.7)	0.063
Respiratory complications	117 (13.8)	89 (21.2)	0.001	133 (13.7)	73 (24.9)	<0.001	106 (13.7)	100 (20.4)	0.002
KDIGO ≥ 2	30 (3.6)	21 (5.0)	0.279	35 (3.6)	16 (5.5)	0.212	24 (3.1)	27 (5.5)	0.048
Wound complications	55 (6.5)	24 (5.7)	0.670	56 (5.8)	23 (7.9)	0.247	50 (6.5)	29 (5.9)	0.793
Composite complications	134 (15.9)	99 (23.6)	0.001	149 (15.3)	84 (28.7)	<0.001	112 (14.5)	121 (24.7)	<0.001
90-day death	20 (2.4)	20 (4.8)	0.034	22 (2.3)	18 (6.1)	0.002	15 (1.9)	25 (5.1)	0.003
1-year death	78 (9.2)	96 (22.9)	<0.001	98 (10.1)	76 (25.9)	<0.001	65 (8.4)	109 (22.2)	<0.001

Data are expressed as number of patients (%) or median [first-third quartiles]. The *p* values represent the group difference between normal and malnutrition within each index. CONUT, controlling nutritional status; GNRI, geriatric nutritional risk index; PNI, prognostic nutritional index; ICU, intensive care unit; MACCE, major adverse cerebro-cardiovascular events; KDIGO, Kidney Disease Improving Global Outcomes classification.

**Table 3:** Impact of preoperative nutritional status on overall and recurrence-free survival after surgery.

	Overall Survival		Recurrence-Free Survival	
Nutritional Index	HR (95% CI) *	*p* for Trend	HR (95% CI) †	*p* for Trend
CONUT	1.23 (1.140–1.34)	<0.001	1.18 (1.10–1.28)	<0.001
No risk (0–1)	reference		reference	
Low risk (2)	1.17 (0.92–1.48)		1.15 (0.92–1.44)	
Moderate risk (3–4)	1.55 (1.24–1.92) ****		1.40 (1.45–1.72) ***	
High risk (≥5)	1.91 (1.47–2.48) ****		1.65 (1.29–2.11) ****	
GNRI	1.28 (1.13–1.45)	<0.001	1.21 (1.08–1.37)	0.001
No risk (>98)	reference		reference	
Low risk (92 to ≤98)	1.23 (0.97–1.56)		1.06 (0.85–1.32)	
Moderate risk (82 to <92)	1.61 (1.22–2.12) ***		1.38 (1.07–1.79) **	
High risk (<82)	2.54 (1.64–3.93) ****		2.03 (1.33–3.09) ***	
PNI	1.27 (1.17–1.38)	<0.001	1.21 (1.12–1.30)	<0.001
No risk (>50)	reference		reference	
Low risk (44.16 to ≤50)	1.58 (1.23–2.03) ****		1.44 (1.15–1.81) ***	
Moderate risk (42 to <44.16)	1.65 (1.20–2.26) ***		1.40 (1.05–1.88) **	
High risk (<42)	2.32 (1.77–3.06) ****		1.90 (1.48–2.44) ****	

*: adjusted by age, pathologic stage of cancer, body mass index, preoperative smoking, preoperative serum uric acid levels, preoperative pulmonary function test (% predicted forced vital capacity), preoperative use of oral hypoglycemic agent, anesthesia time, immediate postoperative hematocrit levels, postoperative SOFAc score, and use of pRBC. †: adjusted by age, pathologic stage of cancer, body mass index, preoperative serum uric acid levels, preoperative use of oral hypoglycemic agent, anesthesia time, immediate postoperative hematocrit levels, postoperative SOFAc score, and use of pRBC. **: *p* value < 0.05; ***: *p* value < 0.01; ****: *p* value < 0.001. *p* for trend was tested for linear trend of HR, and the nutritional indexes (CONUT, GNRI, PNI) were analyzed as if they were continuous variables in the Cox model. HR, hazard ratio; CI, confidence interval; BMI, body mass index; CONUT, controlling nutritional status; GNRI, geriatric nutritional risk index; PNI, prognostic nutritional index; SOFAc, cardiovascular sequential organ failure assessment in the first 24 h; pRBC, packed red blood cell used intraoperatively and postoperatively.

## Data Availability

The dataset used and/or analyzed during the current study is available from the corresponding author on reasonable request.

## Supplementary Materials

The following are available online at https://www.mdpi.com/article/10.3390/nu13114086/s1, Table S1: Impact of preoperative nutritional status on 30-day composite complications after surgery, Table S2: Final multivariable model with nutritional risk groups based on the CONUT score for overall survival, Table S3: Final multivariable model with nutritional risk groups based on the GNRI for overall survival, Table S4: Final multivariable model with nutritional risk groups based on the PNI for overall survival.
