# Supplementary material for: Comparison of Preoperative Nutritional Indexes for Outcomes after Primary Esophageal Surgery for Esophageal Squamous Cell Carcinoma
Source: Nutrients. 2021 Nov 15;13(11):4086. doi: 10.3390/nu13114086 (PMC8619324; doi:10.3390/nu13114086)
Supplement: Supplementary file 1 [file nutrients-13-04086-s001.zip › nutrients-1387386-supplementary.pdf]

-Supplementary Materials-

Comparison of Preoperative Nutritional Indexes for Outcomes after Primary Esophageal Surgery for Esophageal Squamous Cell Carcinoma: A Retrospective Cohort Study

Table of Contents

Table S1. Impact of preoperative nutritional status on 30-day composite complications after surgery

Table S2. Final multivariable model with nutritional risk groups based on the CONUT score for overall survival.

Table S3. Final multivariable model with nutritional risk groups based on the GNRI for overall survival.

Table S4. Final multivariable model with nutritional risk groups based on the PNI for overall survival.

Table S1. Impact of preoperative nutritional status on 30-day composite complications after surgery

	30-day composite complications	
Nutritional index	Odds Ratio (95% Confidence Interval)*	*P* value
CONUT		
Malnutrition (≥ 3)	1.23 (0.88–1.72)	0.227
No risk (0–1)	–	0.603
Low risk (2)	0.95 (0.62–1.47)	0.820
Moderate risk (3–4)	1.15 (0.77–1.72)	0.486
High risk (≥ 5)	1.34 (0.81–2.22)	0.252
GNRI		
Malnutrition (< 92)	1.72 (1.22–2.43)	0.002
No risk (> 98)	–	0.008
Low risk (92 to ≤ 98)	1.38 (0.94–2.03)	0.104
Moderate risk (82 to < 92)	1.90 (1.28–2.83)	0.001
High risk (< 82)	2.10 (1.06–4.17)	0.034
PNI		
Malnutrition (< 44.16)	1.46 (1.03–2.07)	0.033
No risk (> 50)	–	0.163
Low risk (44.16 to ≤ 50)	1.19 (0.77–1.85)	0.440
Moderate risk (42 to < 44.16)	1.59 (0.92–2.74)	0.099
High risk (< 42)	1.67 (1.02–2.74)	0.042

*: adjusted by preoperative chemo-radiation therapy, preoperative serum uric acid levels, preoperative pulmonary function test (% predicted forced vital capacity), anesthesia time, immediate postoperative weight gain (%), and use of pRBC.

CONUT, controlling nutritional status; GNRI, geriatric nutritional risk index; PNI, prognostic nutritional index; pRBC, packed red blood cell used intraoperatively and postoperatively.

Table S2. Final multivariable model with nutritional risk groups based on the CONUT score for overall survival.

Variables	Hazard Ratio (95% Confidence Interval)	*P* value
CONUT		
No risk (0–1)	–	< 0.001
Low risk (2)	1.17 (0.92–1.48)	0.204
Moderate risk (3–4)	1.55 (1.24–1.92)	< 0.001
High risk (≥ 5)	1.91 (1.47–2.48)	< 0.001
Age (yr)	1.02 (1.01–1.03)	0.008
Body mass index (kg/m^2)	0.94 (0.91–0.97)	< 0.001
Pathologic stage of cancer	–	< 0.001
Stage 1	1.03 (0.80–1.32)	0.817
Stage 2	2.23 (1.73–2.88)	< 0.001
Stage 3	3.92 (3.01–5.11)	< 0.001
Stage 4	3.60 (1.85–7.01)	< 0.001
Smoking status		
Non-smoking	–	0.003
Ex-smoking	1.29 (1.05–1.59)	0.014
Current smoking	1.55 (1.20–2.00)	0.001
Preoperative serum uric acid (mg/dl)	0.91 (0.86–0.97)	0.003
FVC (% predicted)	0.99 (0.98–0.99)	0.020
Use of oral hypoglycemic agent	1.37 (1.07–1.75)	0.013
Anesthesia time (hr)	1.12 (1.06–1.17)	< 0.001
Immediate postoperative Hct (%)	0.96 (0.94–0.98)	< 0.001
Postoperative SOFAc score	1.14 (1.06–1.21)	< 0.001
Use of pRBC	1.06 (1.03–1.10)	< 0.001

CONUT, controlling nutritional status; FVC, forced vital capacity; Hct, Hematocrit; SOFAc, cardiovascular sequential organ failure assessment in the first 24 hours; pRBC, packed red blood cell used intraoperatively and postoperatively.

Table S3. Final multivariable model with nutritional risk groups based on the GNRI for overall survival.

Variables	Hazard Ratio (95% Confidence Interval)	*P* value
GNRI		
No risk (> 98)	–	< 0.001
Low risk (92 to ≤ 98)	1.23 (0.97–1.56)	0.084
Moderate risk (82 to < 92)	1.61 (1.22–2.12)	0.001
High risk (< 82)	2.54 (1.64–3.93)	< 0.001
Age (yr)	1.02 (1.00–1.03)	0.010
Body mass index (kg/m^2)	0.98 (0.95–1.02)	0.423
Pathologic stage of cancer	–	< 0.001
Stage 1	0.94 (0.74–1.20)	0.626
Stage 2	2.10 (1.63–2.71)	< 0.001
Stage 3	3.71 (2.85–4.85)	< 0.001
Stage 4	3.51 (1.79–6.87)	< 0.001
Smoking status		
Non-smoking	–	0.009
Ex-smoking	1.29 (1.05–1.59)	0.016
Current smoking	1.46 (1.14–1.88)	0.003
Preoperative serum uric acid (mg/dl)	0.92 (0.86–0.97)	0.004
FVC (% predicted)	0.99 (0.98–1.00)	0.049
Use of oral hypoglycemic agent	1.48 (1.16–1.91)	0.002
Anesthesia time (hr)	1.11 (1.06–1.16)	< 0.001
Immediate postoperative Hct (%)	0.96 (0.94–0.98)	< 0.001
Postoperative SOFAc score	1.13 (1.06–1.21)	< 0.001
Use of pRBC	1.06 (1.03–1.09)	< 0.001

GNRI, geriatric nutritional risk index; FVC, forced vital capacity; Hct, Hematocrit; SOFAc, cardiovascular sequential organ failure assessment in the first 24 hours; pRBC, packed red blood cell used intraoperatively and postoperatively.

Table S4. Final multivariable model with nutritional risk groups based on the PNI for overall survival.

Variables	Hazard Ratio (95% Confidence Interval)	*P* value
PNI		
No risk (> 50)	–	< 0.001
Low risk (44.16 to ≤ 50)	1.58 (1.23–2.03)	< 0.001
Moderate risk (42 to < 44.16)	1.65 (1.20–2.26)	0.002
High risk (< 42)	2.32 (1.77–3.06)	< 0.001
Age (yr)	1.02 (1.01–1.03)	0.010
Body mass index (kg/m^2)	0.94 (0.91–0.97)	< 0.001
Pathologic stage of cancer	–	< 0.001
Stage 1	1.06 (0.82–1.36)	0.659
Stage 2	2.32 (1.79–3.00)	< 0.001
Stage 3	4.08 (3.13–5.33)	< 0.001
Stage 4	4.13 (2.11–8.08)	< 0.001
Smoking status		
Non-smoking	–	0.001
Ex-smoking	1.32 (1.07–1.62)	0.009
Current smoking	1.63 (1.26–2.10)	< 0.001
Preoperative serum uric acid (mg/dl)	0.92 (0.86–0.97)	0.004
FVC (% predicted)	0.99 (0.98–1.00)	0.048
Use of oral hypoglycemic agent	1.43 (1.12–1.83)	0.004
Anesthesia time (hr)	1.12 (1.06–1.17)	< 0.001
Immediate postoperative Hct (%)	0.97 (0.95–0.98)	< 0.001
Postoperative SOFAc score	1.14 (1.06–1.22)	< 0.001
Use of pRBC	1.07 (1.04–1.10)	< 0.001

PNI, prognostic nutritional index; FVC, forced vital capacity; Hct, Hematocrit; SOFAc, cardiovascular sequential organ failure assessment in the first 24 hours; pRBC, packed red blood cell used intraoperatively and postoperatively.
